# Chronic granulomatous disease: A multicenter study from the MENA region

**DOI:** 10.70962/jhi.20260014

**Published:** 2026-07-07

**Authors:** Salem Al-Tamemi, Najla Mekki, Fatima Ailal, Raed Alzyoud, Waleed Al-Herz, Maryam Al-Nesf, Nesrine Radwan, Iman Nasr, Musab Al Jabri, Thouraya Kammoun, Assma Dably, Motasem Alsuweiti, Mehdi Adeli, Eiman Abdalla, Monia Ben Khaled, Mohammad Alnoubani, Hamza Alnsour, Imen Ben-Mustapha, Monia Ouederni, Ahmed Aziz Bousfiha, Mohamed-Ridha Barbouche

**Affiliations:** 1 Department of Child Health, Sultan Qaboos University Hospital, University Medical Center, College of Medicine and Health Sciences, Sultan Qaboos University, Muscat, Oman; 2 Laboratory of Transmission, Control and Immunobiology of Infections (LR16IPT02), Institut Pasteur de Tunis, Faculty of Medicine of Tunis, Université Tunis El Manar, Tunis, Tunisia; 3 Department of Pediatrics, Laboratory of Clinical Immunology, Inflammation and Allergy, Faculty of Medicine and Pharmacy of Casablanca, University of Hassan II, Casablanca, Morocco; 4 Department of Pediatric Allergy, Immunology, and Rheumatology, Queen Rania Children’s Hospital, Amman, Jordan; 5 Department of Pediatrics, College of Medicine, Kuwait University, Kuwait City, Kuwait; 6 Allergy and Immunology Division, Department of Medicine, Hamad Medical Corporation, Doha, Qatar; 7 Pediatric Allergy, Immunology & Rheumatology Unit, Ain Shams University, Cairo, Egypt; 8 Department of Clinical Immunology and Allergy, Royal Hospital, Muscat, Oman; 9 Department of Pediatrics, Hedi Chaker University Hospital, Sfax, Tunisia; 10 Laboratory of Clinical Immunology, Inflammation and Allergy Faculty of Medicine and Pharmacy, Hassan II University, Casablanca, Morocco; 11 Faculty of Medicine, University of Tunis El Manar, Tunis, Tunisia; 12 Pediatric Immuno-Hematology Unit, Bone Marrow Transplantation Center Tunis, Tunis, Tunisia; 13 Department of Immunology, Institut Pasteur de Tunis, Tunis-Belvedere, Tunis, Tunisia; 14 Department of Pediatrics, Hassan II University, Laboratory of Clinical Immunology, Inflammation and Allergy, Casablanca, Morocco; 15 Department of Microbiology, Immunology and Infectious Diseases, College of Medicine and Health Sciences, Arabian Gulf University, Manama, Bahrain

## Abstract

To study chronic granulomatous disease (CGD) in the Middle East and North Africa (MENA) region, we conducted a retrospective multicenter study to investigate the clinical features, infections, microorganisms, management, mortality, and survival of patients with CGDs. A total of 322 patients were included; the M:F ratio was 1.8, and the median ages at disease onset and diagnosis were 6.0 and 20.0 mo, respectively. The most common infections included pneumonia (58.0%), lymphadenitis (49.1%), skin abscesses/cellulitis (40.7%), and invasive pulmonary aspergillosis (17.7%). The most commonly isolated microorganisms were *Staphylococcus* spp. (22%), *Aspergillus* spp. (19.3%), and *Pseudomonas* spp. (11.2%)*.* The genetic diagnosis was established in 108 (30.4%) patients: AR inheritance in 84.3% and X-linked in 15.7%. The median survival age is 8.5 years, and the estimated 10-year survival rate is 77.3%. CGD represents a significant disease burden in the MENA region, necessitating early intervention with molecular/genetic testing and HSCT availability.

## Teaser

Al-Tamemi et al. describe that autosomal recessive CGD represents a significant disease in the MENA region associated with significant morbidity and mortality. Regional challenges are related to universal newborn BCG vaccine, lack of newborn screening programs, and limitation of curative interventions such as hematopoietic stem cell transplantation and gene therapy.

## Introduction

Chronic granulomatous disease (CGD) is a known inborn error of immunity (IEI) that affects many patients worldwide. It is a disease of neutrophils, with defects in the production of oxidative burst molecules that are important in the microbial killing of microorganisms, mainly bacteria and fungi (1, 2). The NADPH complex generates an oxidative burst, producing free oxygen radicals and hypochloric acid that are toxic to microorganisms (3). The NADPH complex comprises a chain of several protein subunits extending from the cellular membrane to the cytoplasmic compartment: gp91, p22, p47, p67, and p40. These proteins are essential for the transfer of electrons from the cytoplasm to the membrane upon the activation of phagocytes by IFN-γ, which is secreted by activated T cells as a result of microbial antigens presented by antigen-presenting cells (4, 5).

Molecular defects in any of the NADPH protein subunits are associated with nearly a similar clinical disease characterized by recurrent and severe infections caused by intracellular gram-positive, gram-negative bacteria and fungi such as *Staphylococcus aureus*,* Pseudomonas* spp.,* Nocardia* spp.,* Aspergillus* spp.,* Mycobacterium* spp., and other microorganisms (6, 7, 8, 9, 10). Infections can affect any organ, but the lung is widely affected by pneumonia, which can often be complicated by abscess formation and cavitation. Lymph nodes are usually affected by lymphadenitis secondary to bacterial microorganisms, and bacteria or fungi may affect the liver, brain, spine, and skull. Simple Bacillus Calmette–Guérin (BCG) adenitis or disseminated BCG infections may occur in certain patients after BCG vaccination, a live attenuated vaccine made of bovine mycobacterium (11, 12, 13, 14, 15, 16, 17). Immune dysregulation and hyperinflammation are well-recognized manifestations in some patients with CGD, such as gastrointestinal granulomas, inflammatory colitis, genitourinary granulomas, and autoimmune cytopenia, which complicate the course of the disease (18, 19, 20, 21, 22, 23, 24, 25).

The diagnosis is traditionally established by measuring the oxidative burst via the nitro-blue tetrazolium (NBT) test or, recently, via the dihydrorhodamine 1,2,3 (DHR) test via flow cytometry, with the latter being more sensitive and quantitative in diagnosing patients but also helpful in identifying carrier status (26, 27, 28, 29, 30, 31). Genetic diagnosis is more easily confirmed by testing for targeted known pathogenic variants, gene sequencing, or whole-exome sequencing; pathogenic variants in the genes of the NADPH complex that cause CGD have been reported, and genetic testing is essential for family counseling, early diagnosis after birth, and preimplantation genetic testing (32, 33, 34).

The management of patients with CGD includes appropriate and aggressive treatment of infections and manifestations via the use of broad-spectrum antibacterial and antifungal agents with the aim of identifying the causative microorganisms for targeted therapy. Inflammatory manifestations may necessitate anti-inflammatory and immunosuppressive therapy. Surgical intervention may be essential for removing fluid collection or debridement from affected organs (35). After diagnosis, patients with CGD must be on prophylactic medications such as cotrimoxazole and itraconazole to prevent bacterial and fungal infections, effectively preventing infections when the patient is compliant (36, 37, 38, 39, 40). IFN-γ therapy is used as adjunctive treatment for acute infections and long-term prevention; however, its unavailability and cost have been challenging for most centers (41, 42, 43, 44). The curative treatment for patients with CGD is hematopoietic stem cell transplantation (HSCT) with excellent results when the donor is an HLA-matched sibling. Recently, gene therapy for certain molecular defects has shown promising results and preimplantation genetic testing is being increasingly used. The survival of patients with CGD has improved over the past few decades, mainly because of preventive therapy, improvements in identifying causative organisms and their treatment, and, recently, the availability of curative options (24, 25, 45, 46).

## Results

### Baseline characteristics

**Figure 1.**
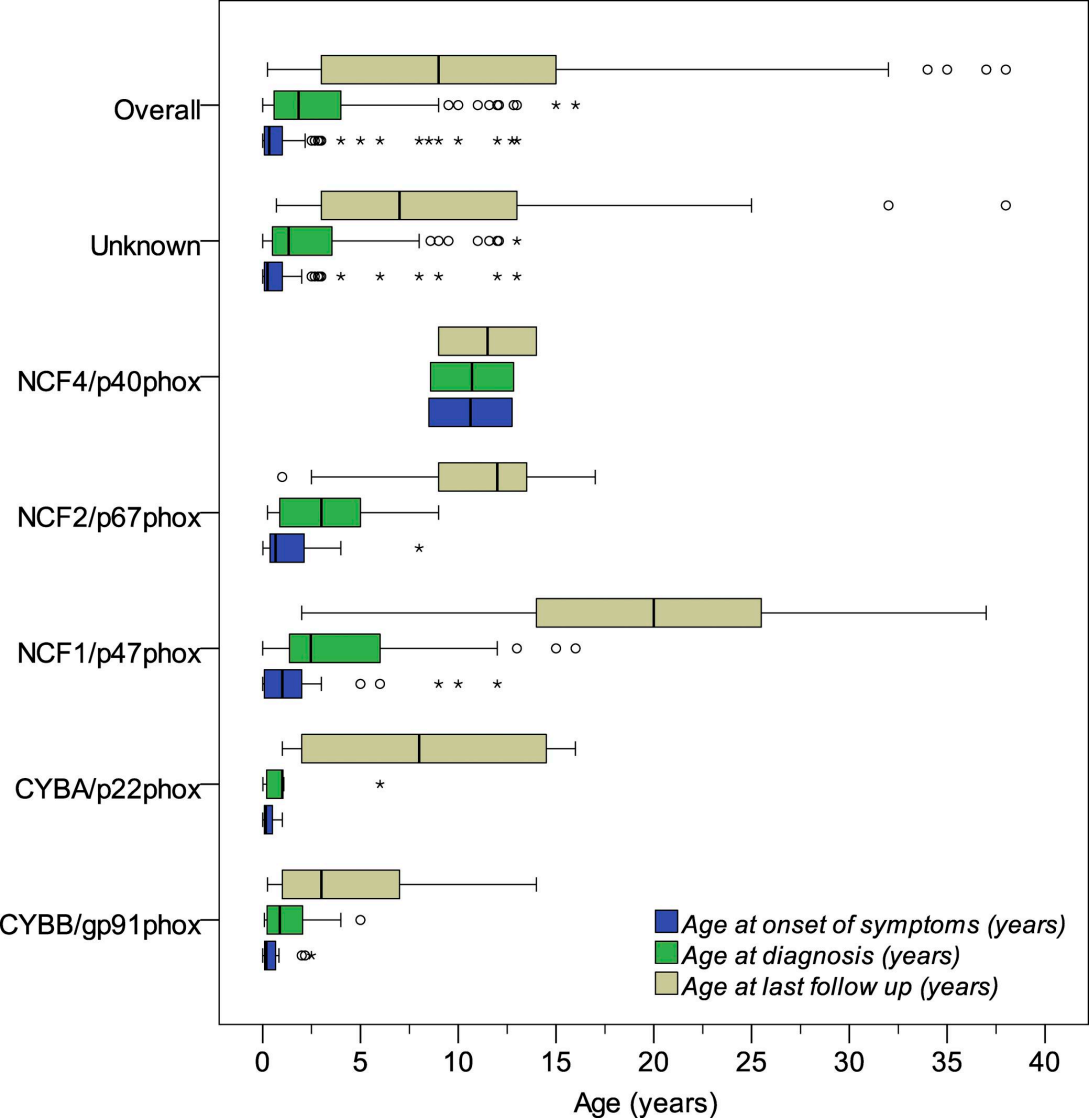
**Boxplot chart illustrates the median and IQR for the ages at the onset of symptoms, at the time of diagnosis, and at the last follow-up for patients with CGDs on the basis of their genotypes, both unknown and overall.** Age at the last follow-up significantly differed between the CGD genotypes (P < 0.005). Mild outliers are indicated by circles and extreme outliers are indicated by asterisks.

The number of patients included in this study was 322 (Table 1), with a male‒female ratio of 1.8 and a male predominance of 64.3%. The mean age of onset of symptoms was 20.2 mo (standard deviation [SD]: 54.7), with a median of 6.0 mo (interquartile range [IQR]: 2.0–18.0) and a range of 0–53 years. The mean age at diagnosis was 40.1 mo (SD: 64.4), the median age at diagnosis was 20.0 mo (IQR: 7.0–48), and the age range was 0–58 years. The overall mean age at the last follow-up was 10.5 years (SD: 9.0), with a median of 8.5 years (IQR 3.0–15.0) and a range of 0.3–63 years (Fig. 1). Age and quartiles for the overall and different genotypes of patients with CGD were included in the study. There was a significant difference between the different genotypes in terms of age at the last follow-up (P < 0.005). A family history of CGD was present in 104 (32.3%) patients, consanguinity in 194 (60.2%) patients, and death of a previous sibling in 72 (22.4%) patients. Routine BCG vaccination was administered to 278 (86.3%) patients.

**Table 1.** Baseline characteristics of patients with CGD in the MENA region

Patients characteristics	Values
Number of patients	322
Male:female	1.8
Age of disease onset	Mean 20.2 (SD: 54.7) mo, median 6.0 (IQR 2.0–18.0) mo, range 0–53 years
Age at diagnosis	Mean 40.1 (SD: 64.4) mo, median 20.0 (IQR: 7.0–48.0) mo, range 0–58 years
Age at last follow-up (survival age)	Mean 10.6 (SD 9.0) years, median 8.5 (IQR: 3.0–15.0) years, range 0.3–63.0 years
Family history of CGD	104 (32.3%)
Consanguinity	194 (60.2%)
Early diagnosis after birth	55 (17.1%)
Death of a previous sibling	72 (22.4%)
Genetic diagnosis	98 (30.4%)
HSCT	38 (11.8%)
Mortality	53 (16.5%)
BCG vaccine	278 (86.3%)
Survival (10-year)	Overall 77.3%
	AR-CGD 88.0%, XL-CGD 49.6%

HSCT, hematopoietic stem cell transplantation; AR-CGD, autosomal recessive chronic granulomatous disease; XL-CGD, X-linked chronic granulomatous disease; BCG, bacillus Calmette–Guérin.

### Clinical features

The types of infections developed by the patients with CGD are shown in Table 2: BCG adenitis in 92 (28.6%) patients, disseminated BCG infection in 39 (12.1%) patients, pneumonia in 189 (58.7%) patients, bacterial pneumonia in 122 (37.8%) patients, mycobacterial pneumonia in 28 (8.7%) patients, fungal pneumonia in 63 (19.6%) patients, invasive pulmonary aspergillosis in 57 (17.7%) patients, bacterial lymphadenitis in 189 (58.7%) patients, skin abscess/cellulitis in 131 (40.9%) patients, bacteremia/septicemia in 65 (20.2%) patients, lung abscess in 37 (11.5%) patients, liver abscess in 33 (10.2%) patients, brain abscess in 13 (4.0%) patients, osteomyelitis/septic arthritis in 33 (10.2%) patients, and meningitis/encephalitis in 30 (9.3%) patients.

**Table 2.** Clinical infections and immune dysregulation in patients with CGD in the MENA region

Infections	Number (%)
Pneumonia	189 (58.7%)
Bacterial lymphadenitis	158 (49.1%)
Skin abscess/cellulitis	131 (40.7%)
Bacterial pneumonia	122 (37.9%)
BCG adenitis	92 (28.6%)
Bacteremia/septicemia	65 (20.2%)
Fungal pneumonia	63 (19.6%)
Invasive pulmonary aspergillosis	57 (17.7%)
Disseminated BCG infection	39 (12.1%)
Lung abscess	37 (11.5%)
Liver abscess	33 (10.2%)
Osteomyelitis/septic arthritis	33 (10.2%)
Meningitis/encephalitis	30 (9.3%)
Mycobacterial pneumonia	28 (8.7%)
Brain abscess	13 (4.0%)

Immune dysregulation	Number (%)
Autoimmune hemolytic anemia	54 (16.8%)
Allergic disease	31 (9.6%)
Immune thrombocytopenic purpura	20 (6.2%)
Inflammatory colitis	18 (5.6%)
Urogenital obstruction	15 (4.7%)
Systemic lupus erythematosus	5 (1.6%)
Skin granuloma	5 (1.6%)
GIT granuloma	4 (1.2%)
Lung granuloma	3 (0.9%)
Celiac disease	3 (0.9%)
Rheumatoid arthritis	2 (0.6%)
Hypothyroidism	2 (0.6%)
Sarcoidosis	2 (0.6%)
Myasthenia gravis	1 (0.3%)
Malignancy	1 (0.3%)

BCG, bacillus Calmette–Guérin; GIT, gastrointestinal tract.

**Figure 2.**
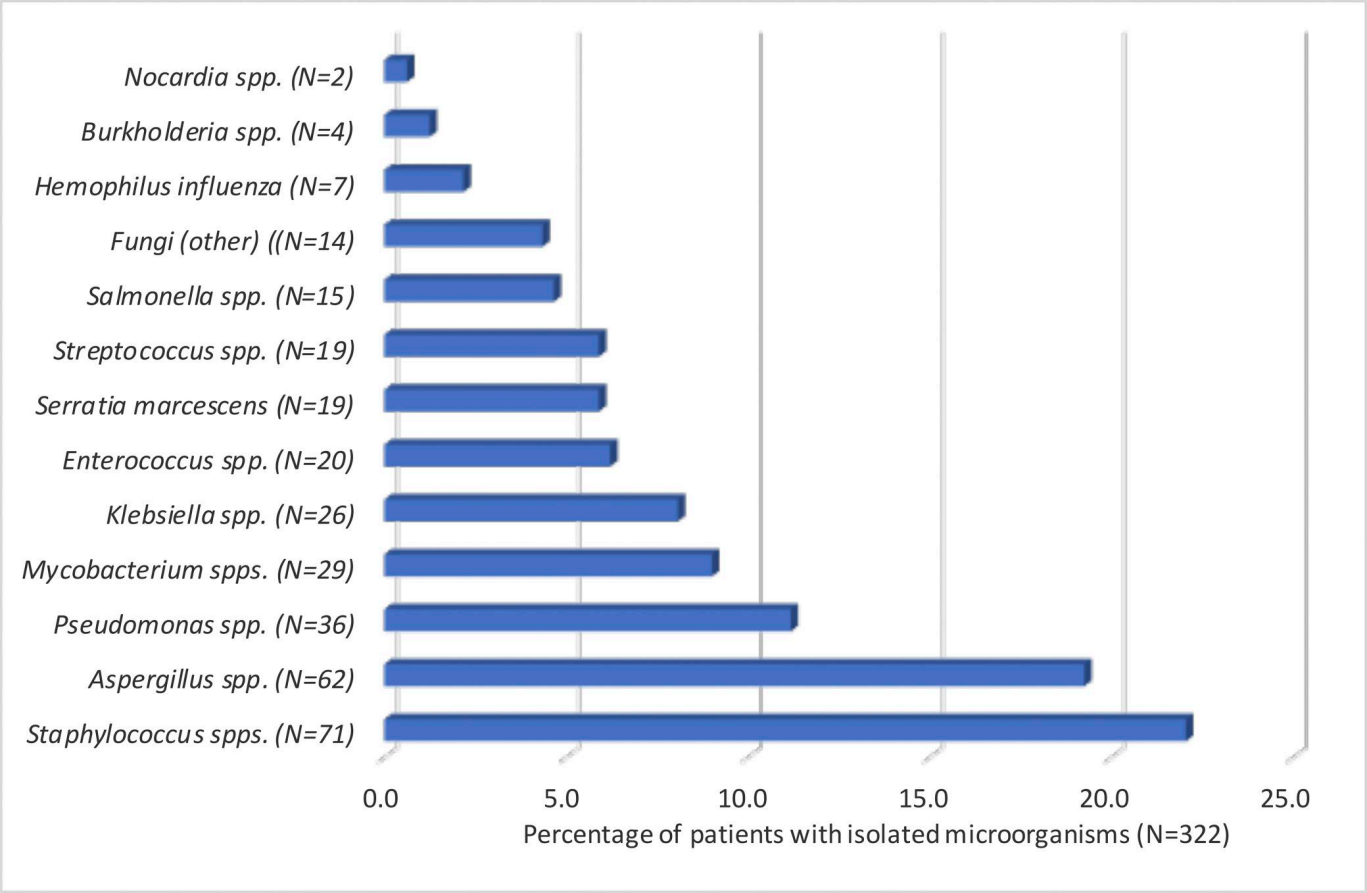
**T** **he microorganisms isolated from patients with CGD.**

The isolated microorganisms (Fig. 2) were *Staphylococcus* spp. in 71 (22.0%), *Aspergillus* spp. in 62 (19.3%),* Pseudomonas* spp. in 36 (11.2%),* Mycobacterium* spp. in 29 (9.0%),* Klebsiella* spp. in 26 (8.1%),* Enterococcus* spp. in 20 (6.2%),* Serratia marcescens* in 19 (5.9%),* Streptococcus* spp. in 19 (5.9%),* Salmonella* spp. in 15 (4.7%), other fungi in 14 (4.3%),* Haemophilus influenzae* in 7 (2.2%),* Burkholderia* spp. in 4 (1.2%), and* Nocardia* spp. in 2 (0.6%) patients (Fig. 2).

Immune dysregulation affected a significant number of patients (Table 2): autoimmune hemolytic anemia in 54 (16.8%) patients, allergic diseases in 31 (9.6%) patients, immune thrombocytopenia in 20 (6.2) patients, inflammatory colitis in 18 (5.6%) patients, urogenital obstruction in 15 (4.7%) patients, systemic lupus erythematosus in 5 (1.6%) patients, skin granuloma in 5 (1.6%) patients, intestinal granuloma in 4 (1.2%) patients, lung granuloma in 3 (0.9%) patients, and celiac disease in 3 (0.9%) patients. The long-term complications in the presented patients were as follows: combined restrictive and obstructive lung disease in 29 (9.0%) patients, bronchiectasis in 21 (6.5%) patients, pulmonary fibrosis in 17 (5.3%) patients, and heart disease in 9 (2.8%) patients (valvular heart disease and right-sided heart failure).

### Diagnosis & management

All patients were diagnosed with CGD via either the NBT test or the DHR test. Genetic diagnosis was established in 98 (30.4%) patients as follows: 44 (44.9%) had NCF1/p47phox, 27 (27.6%) had NCF2/p67phox, 16 (16.3%) had CYBB/gp91phox, 9 (9.2%) had CYBA/p22phox, and 2 (2.0%) had NCF4/p40phox. The following pathogenic variants were commonly reported from Oman: NM_000265.6: c.579G > A p. (Trp193Ter) in the NCF1 gene encoding p47phox in 23 of 31 (74.2%) patients, c.1320C>G; p(Tyr440*) in CYBB/gp91phox in 4 of 31 (12.9%) patients, and G784A Gly262Ser in NCF1/p47phox in 4 of 31 (12.9%) patients; Tunisia: c.257+2T>C (A59IfsX2) in the NCF2/p67phox in 11 of 20 (55%) patients and c.75_76delGT in the NCF1/p47phox in 5 of 20 (25%) patients; and Jordan: c.1171_1175delAAGCT in the NCF2-Exon 12 p. Lys391Glufs*9 PB1 domain in the NCF2/p67phox in 8 of 18 (44.4%) patients.

The inheritance pattern was identified as autosomal recessive (AR) in 91 of 108 (84.3%) patients and X-linked (XL) in 17 of 108 (15.7%) patients. Prophylactic cotrimoxazole was administered to 216 (67.1%) patients, itraconazole was used in 224 (69.6%) patients, IFN-γ therapy was used for acute infections in 45 (14.0%) patients, IFN-γ long-term prophylaxis was used in 7 (2.2%) patients, and one patient received a granulocyte transfusion during infection. HSCT was performed in 38 (11.8%) patients.

### Mortality, survival, and causes of death

The overall mortality rate at the end of the study was 53 patients (16.5%). It affected 22% of patients with AR-CGD and 64.7% of patients with XL-CGD (P = 0.005). The overall mean survival age was 10.6 years (SD: 9.0), the median survival age was 9.0 (IQR: 3.0–15.0), the range was 0.3–63; the AR-CGD mean was 14.9 years (SD: 8.9), the median was 14.0 (IQR: 8.5–20.0), the range was 1.0–37; the XL-CGD mean was 4.5 years (SD: 4.0), the median was 4.0 (IQR: 1.0–7.5), the range was 0.3–14.0; the NCF1/p47phox mean was 19.3 years (SD: 8.6), the median was 20.0 (IQR: 14.0–25.8), the range was 2.0–37.0; the NCF2/p67 mean was 10.6 years (SD: 4.8), the median was 12.0 (IQR: 9.0–14.0), the range was 1.0–17.0; and the CYBA/p22phox mean was 8.3 years (SD: 6.7).

**Figure 3.**
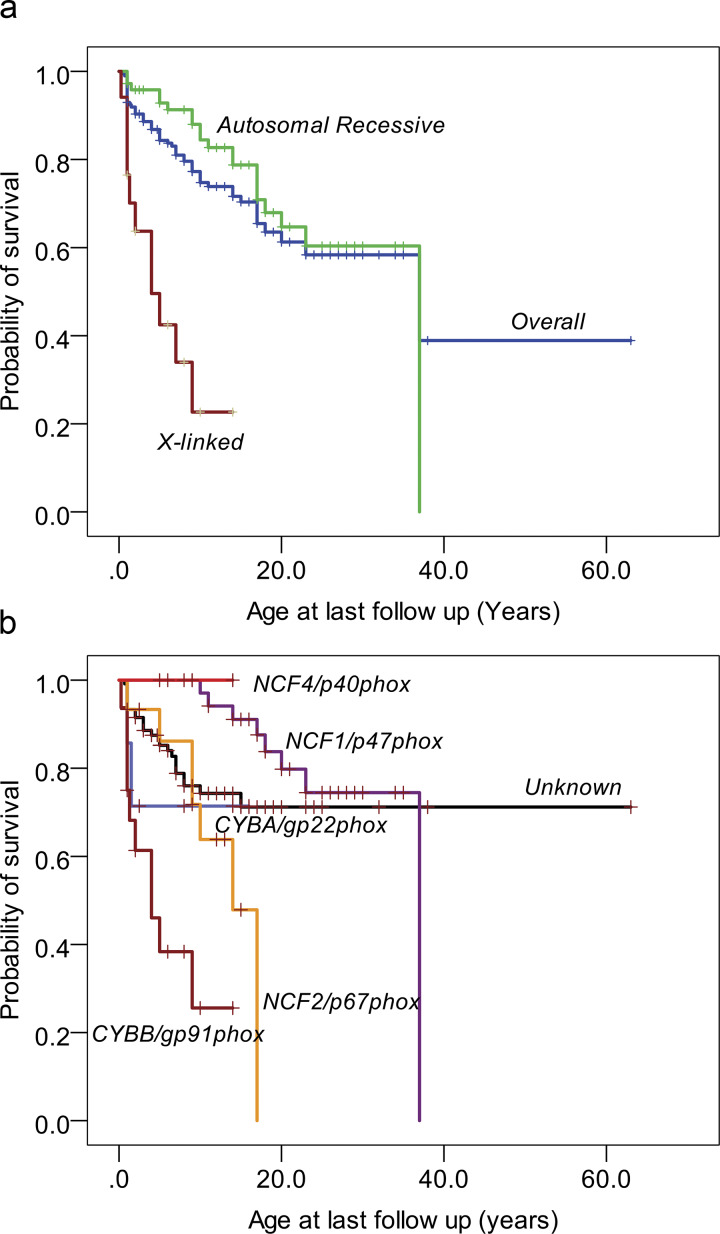
**Kaplan‒Meier curve illustrates the probability of survival. (a)** For XL-CGD, AR-CGD, and overall survival, survival is based on the age of the last follow-up. **(b)** Different genotypes of CGD. Log-rank (Mantel‒Cox), Breslow (generalized Wilcoxon), and Tarone‒Ware tests, P < 0.005.

The estimated 10-year overall survival rate based on the age of the last follow-up was 77.3%, that of AR-CGD was 88.0%, that of XL-CGD was 49.6%, that of NCF1/p47phox was 97.1%, that of NCF2/p67 was 63.8%, that of CYBA/p22phox was 71.4%, and that of CYBB/gp91phox was 25.6% (P = 0.005) (Fig. 3). The survival of patients who received HSCT was 82.4% in the AR-CGD group and 66.7% in the XL-CGD group. Among the causes of death in the study patients were septicemia, severe pulmonary disease and acute respiratory distress syndrome, invasive fungal disease, disseminated mycobacterial disease, and deep abscess formation involving the lung, liver, spleen, and brain.

## Discussion

This study is the first multicenter study from the Middle East and North Africa (MENA) region to describe 322 patients with CGD (Table 3). The main findings of this study are compared with those of studies from the USA and Europe (47, 48). Males are nearly twice as affected as females are, which could be explained by multiple molecular causes of CGD and founder mutations in particular populations. The median onset of symptoms and diagnosis highlights the early presentation and relatively early diagnosis in most patients; however, one patient who presented at the age of 53 years highlighted that CGD is a variable disease and should be considered in adults with unusual infections. CGD presenting in adulthood has been reported previously by Winkelstein et al. (47).

**Table 3.** Clinical features of patients with CGD from the MENA region compared with those from the USA and Europe

​	MENA (2025)	USA (2000) (47)	Europe (2009) (48)
Number of patients	322	368	429
Sex	Males 64.3%	Males 85.9%	Males 81.8%
Age at onset of symptoms	Mean 20.2 mo Median 6.0 mo	N/A	N/A
Age at diagnosis	Mean 40.1 mo Median 20.0 mo	Mean XL-CGD 3.0 years AR-CGD 7.8 years	Mean XL-CGD 4.9 years AR 8.8 years
Diagnosis delay	17 mo	N/A	N/A
Consanguinity	60.2%	N/A	N/A
CGD diagnosis	Genetic diagnosis 30.4% XL-CGD 15.7% AR-CGD 84.3%	XL-CGD 69.8% AR-CGD 22%	XL-CGD 67% AR-CGD 33%
Genotype	gp91phox 16/98 (16.3%) p47phox 44/98 (44.9%) p67phox 27/98 (27.6%) p22phox 9/98 (9.2%) p40phox 2/98 (2.0%)	gp91phox 259/368 (70.4%) p47phox 45/81 (56%) p67phox 10/81 (12%) p22phox 7/81 (8%)	gp91phox 290 (67%) p47phox 69/139 (49%) p67phox 11/139 (8%) p22phox 22/139 (16%)
Common infections	Pneumonia 58.7% Lymphadenitis 49% Skin abscess 41%	Pneumonia 79% Abscess 68% Adenitis 53%	Lung 66% Skin 53% Lymph nodes 50%
Common microorganisms	BCG adenitis 28.6% *Staphylococcus* spp. 22% *Aspergillus* spp. 19.3% Disseminated BCG 12.1%	*Aspergillus* spp. 41% *Staphylococcus* spp. 41.2% *Serratia* spp. 24.5%	*S. aureus* 30% *Aspergillus* spp. 26% *Salmonella* spp. 16% BCGitis 8%
Immune dysregulation	AIHA 16.8% ITP 6.2% Colitis 5.6% SLE 1.6%	Lupus syndromes 3.3% Colitis 17.4% Granuloma 26.4% ITP 1.4%	N/A
Management	Cotrimoxazole 67% Itraconazole 69.6% IFN-g 14% Granulocyte Tx. 1 patient HSCT 11.8%	Antibiotics 89% IFN-g 73% Granulocyte Tx. 15% HSCT 2 patients	Antibiotics 71% Antifungal 53% IFN-g 33% Granulocyte Tx. 7% HSCT 6%
Mortality and survival	16.5% **Median survival age** XL-CGD 4.0 years AR-CGD 14.0 years **10-year survival** XL-CGD 49.6% AR-CGD 88.0%	17.6% **5-year follow-up** XL-CGD 76% AR-CGD 88%	20% **Median death age** XL-CGD 8.8 years AR-CGD 10.4 years

AR-CGD, autosomal recessive chronic granulomatous disease; XL-CGD, X-linked chronic granulomatous disease; BCG, bacillus Calmette–Guérin; AIHA, autoimmune hemolytic anemia; ITP, immune thrombocytopenic purpura; SLE, systemic lupus erythematosus; IFN-g, interferon γ; Tx., transfusion; HSCT, hematopoietic stem cell transplantation.

Although there was no significant difference between CGD subtypes in terms of the onset of symptoms and diagnosis, there was a considerable difference between subtypes in terms of age at the last follow-up, indicating different severities. One third of our patients had a positive family history; this is important for screening, early diagnosis, and avoidance of the BCG vaccine in newborns pending screening for the disease. Since consanguineous marriages are customary in the MENA region, nearly two thirds of our patients are born to consanguineous parents (49, 50). This consanguinity explains the predominance of autosomal recessive inherited CGD genes among our patients in contrast to the X-linked genes reported from Europe and the USA (47, 51, 52).

The clinical features and microorganisms identified in this study are similar to those reported in other studies: pneumonia, lymphadenitis, skin abscesses, *Staphylococcus *spp., *Aspergillus* spp.,* Pseudomonas* spp.,* Mycobacterium* spp., and *Klebsiella* spp. However, a significant number of patients developed BCG adenitis and BCG vaccine–associated disseminated infection. BCG is a live attenuated vaccine recommended by the World Health Organization universally in endemic countries to battle against *Mycobacterium tuberculosis*, particularly tuberculosis (TB) meningitis and miliary TB, the two deadly complications of TB (53). BCG vaccine–associated disseminated infection is challenging to treat and can be fatal, contributing to morbidity and mortality in patients from regions other than the USA and other countries where BCG is not a routine vaccine (54).

Infections with *Aspergillus* spp. and *Staphylococcus *spp. stand out as a significant burden in patients with CGD, as evidenced by our study and studies from the USA and Europe. Surprisingly, *Nocardia* and *Burkholderia* were reported in only a few patients; this could be due to challenges in isolating these organisms. In this study, immune dysregulation and inflammation manifestations were not frequent compared with those reported in the USA study, where more patients reported developing colitis, which could be attributed to the predominance of XL-CGD. Following recurrent pneumonia and its complications, some patients develop bronchiectasis and fibrosis, resulting mainly in restrictive lung disease but also in combined obstructive and restrictive disease.

Diagnosing CGD is now considered straightforward using the DHR assay, which is available at all participating centers, though the NBT test was historically used for this purpose. Although genetic testing is not necessary for diagnosis, it is essential for prognostic reasons and family counseling and for preimplantation genetic testing. Only one third of our patients received a genetic diagnosis, which is likely due to the unavailability of molecular and genetic facilities throughout the study period; however, we are seeing an increase in molecular and genetic diagnoses for inherited disorders in general. This should aid in premarital genetic screening, identification of CGD carriers, and counseling, and hence a reduction in disease incidence; this is in contrast to the findings of studies in the USA and Europe, where a molecular/genetic diagnosis was established in >90% of patients and used to subtype CGD in addition to genotyping (47, 48). Different founder variants have been reported previously from different areas in the region, and this has facilitated early diagnosis and premarital counseling (32, 34, 55, 56, 57).

Adherence to prophylactic cotrimoxazole and itraconazole remains a challenge, as only two thirds of patients were reported to be on treatment, which is similar to the findings of studies in the USA and Europe; from our clinical observations, patients who were not adherent to these medications develop invasive infections that may be fatal. Despite challenges in terms of the availability and cost of IFN-γ, it was used in fewer patients in our study than in the USA and Europe did. Worldwide, more patients with CGD are surviving to adulthood because of improvements in diagnosis, microorganism identification, and antimicrobial use. Therefore, noninfectious complications have become increasingly recognized. Moreover, the number of patients with CGD who have received HSCT with excellent success has increased (24, 58). Approximately 12% of the patients in this study underwent transplantation, which is greater than that reported in studies in the USA and Europe; this could be because of the decades-long difference between the studies, and HSCT is increasingly accepted as a standard treatment option for patients with CGD.

Although the number of XL-CGD patients in our study was smaller, the mortality rate at the end of the study was comparable to that of the USA study, which was significantly greater in the XL-CGD group than in the AR-CGD group. Additionally, there was a similar significant difference in the median survival age of patients with different genetic subtypes of CGD because of the difference in severity among different genotypes. Similar studies reported differences in severity between the two inheritance patterns (59, 60). The difference in survival among the AR-CGD and the XL-CGD genotypes could be related to the residual activity of NADPH oxidase, which has been shown previously to be correlated with disease severity and survival (61). Hence, it is important to perform a genetic diagnosis in patients with abnormal oxidative bursts. Similarly, the estimated probability of survival is variable for different CGD genotypes, as illustrated in Fig. 3. This is important when patients are counseled and when major decisions such as HSCT, including prioritizations, are made. Emerging gene therapy for certain genotypes of patients with CGD has been reported; however, this modality is not yet available in the region (62). Preimplantation genetic testing for various genetic disorders is available and relatively new in the region and should be an option for families with a diagnosis of CGD, with an additional advantage of HLA typing for a future donor of an affected child (63, 64, 65).

### Conclusion

This study revealed that CGD represents a significant IEI in the MENA region, with a particular challenge of BCG-related complications in certain patients, as there is no newborn screening for CGD. However, this could be overcome by avoiding BCG in newborns if a family history is present or if there is a sibling death pending investigation. Improving adherence to prophylactic treatment, the management of severe infection, complications, and the feasibility of HSCT should improve the survival of patients with CGD in the region. The variability in genotype severity and survival makes obtaining a molecular/genetic diagnosis necessary to help prevent new patients through family counseling, preimplantation genetic diagnosis, and the future development of gene therapy.

## Materials and methods

### Study design

This study was a retrospective multicenter study from the MENA region, an initiative of the Arabic Association for Primary Immunodeficiency. The aim of this study was to describe the clinical features, infections, diagnosis, treatment, and survival of patients with CGD and to compare them with those of major studies from Europe and the USA. The participating centers were from the following countries: Oman, Qatar, Kuwait, Jordan, Egypt, Tunisia, and Morocco. The investigators who participated in this study were immunologists who cared for patients with CGD.

### Subjects and data collection

This study included both male and female patients with a diagnosis of CGD at the time of data collection in the year 2025, including pediatric and adult age groups. The patients were diagnosed at different times between the years of 1983 and 2025 and followed up for variable durations of time by the participating investigators in the respective centers and received a standard of care for CGD by the respective centers. Patients with other forms of immunodeficiency and phagocytic disorders were excluded. The total number of patients for whom data were available was 322, and the age range was 0–58 years. The data were collected by using an electronic form that was sent to the participating investigators and included variables such as age; sex; age of onset of symptoms; age at diagnosis; consanguinity; family history; previous death of siblings; BCG vaccination; BCG infection; the presence of pneumonia; fungal pneumonia; lung, liver, and brain abscess; inflammatory manifestations such as colitis; immune dysregulation, such as immune thrombocytopenia; hemolytic anemia; systemic lupus erythematosus; isolated microorganisms; diagnostic methods for CGD; genetic diagnosis; treatment; duration of follow-up; and survival. The diagnosis of CGD was made on the basis of an abnormal NBT or DHR test or a pathogenic variant in CGD-causing genes according to the International Union of Immunological Societies classification (66). The collected data were transferred into an Excel sheet and then exported to the Statistical Package for the Social Sciences version 23 for analysis.

### Statistical analysis

The data of all 322 patients were used for the analysis. Analysis was performed by using the following tools: frequencies, percentages, means, SDs, medians, IQRs, and ranges. Statistical associations were analyzed via Pearson’s chi-square test for categorical variables and ANOVA for continuous variables. Spearman’s rank correlation was used to examine the correlation between ranked variables. The Kaplan‒Meier method was used to analyze the overall survival and survival of patients with different subtypes of CGD. A P value <0.05 was considered to indicate statistical significance.

## Ethics approval

The study was conducted in accordance with the principles of the Declaration of Helsinki.

## Consent to participate

The authors affirm that informed consent was obtained from all patients prior to the investigations and interventions. Consent for genetic testing and interventions was obtained from patients by participating investigators at the respective centers. The Qatar data were part of the Qatar National Primary Immunodeficiency Disease Registry, MRC-01-25-151, Institution Review Board 16299/16.

## Consent to publish

The authors affirm that no identifiable patient data were published in any form.

## Data Availability

Datasets generated during and analyzed during the current study are available from the corresponding author upon request.
